# Inflammatory and Oxidative Stress Responses of an Alveolar Epithelial Cell Line to Airborne Zinc Oxide Nanoparticles at the Air-Liquid Interface: A Comparison with Conventional, Submerged Cell-Culture Conditions

**DOI:** 10.1155/2013/652632

**Published:** 2013-01-02

**Authors:** Anke-Gabriele Lenz, Erwin Karg, Ellen Brendel, Helga Hinze-Heyn, Konrad L. Maier, Oliver Eickelberg, Tobias Stoeger, Otmar Schmid

**Affiliations:** ^1^Comprehensive Pneumology Center, Institute of Lung Biology and Disease, Helmholtz Zentrum München, Ingolstaedter Landstrasse 1, 85758 Neuherberg, Germany; ^2^Joint Mass Spectrometry Center, Helmholtz Zentrum München, Ingolstaedter LandstraBe 1, 85758 Neuherberg, Germany

## Abstract

The biological effects of inhalable nanoparticles have been widely studied *in vitro* with pulmonary cells cultured under submerged and air-liquid interface (ALI) conditions. Submerged exposures are experimentally simpler, but ALI exposures are physiologically more realistic and hence potentially biologically more meaningful. In this study, we investigated the cellular response of human alveolar epithelial-like cells (A549) to airborne agglomerates of zinc oxide (ZnO) nanoparticles at the ALI, compared it to the response under submerged culture conditions, and provided a quantitative comparison with the literature data on different types of particles and cells. For ZnO nanoparticle doses of 0.7 and 2.5 **μ**g ZnO/cm^2^ (or 0.09 and 0.33 cm^2^ ZnO/cm^2^), cell viability was not mitigated and no significant effects on the transcript levels of oxidative stress markers (HMOX1, SOD-2 and GCS) were observed. However, the transcript levels of proinflammatory markers (IL-8, IL-6, and GM-CSF) were induced to higher levels under ALI conditions. This is consistent with the literature data and it suggests that *in vitro* toxicity screening of nanoparticles with ALI cell culture systems may produce less false negative results than screening with submerged cell cultures. However, the database is currently too scarce to draw a definite conclusion on this issue.

## 1. Introduction

Exposure to airborne particles has been linked to adverse health effects including pulmonary inflammation, thrombosis, neurodegeneration, and cardiovascular disease [1–3]. A number of studies have indicated that particles with diameters below 100 nm have a more pronounced effect than larger particles, implying that nanoparticles (or ultrafine particles) are more toxic on a mass basis [3–6]. 

 Zinc is an ubiquitous transition metal associated with industrial emissions (e.g., mining and smelting of zinc) that typically appears in the form of zinc oxide (ZnO) in ambient particulate matter (PM) [7–9]. ZnO is known as an occupational hazard, since inhalation of high concentrations of ZnO formed during welding activities can lead to metal fume fever [10, 11] associated with a marked upregulation of proinflammatory markers in the lung [11–13]. In addition to these inadvertently generated ZnO nanoparticles, there is a variety of ZnO nanostructures, which have shown great potential for nanotechnological products including manufacturing and pharmaceutical applications [14, 15]. However, there is increasing concern that the desirable technological characteristics of nanosized ZnO may be countervailed by increased health and environmental risks due to toxic effects that do not occur for bulk ZnO. While the enhanced toxicity potential of nanoparticles is at least in part due to their inherently large surface-to-mass ratio [4, 6, 16, 17], there is also evidence that some metal particles trigger additional toxicological pathways making them more toxic (per surface area) than many other particle types (e.g., carbon, polystyrene) [18]. 

**Figure 1:**
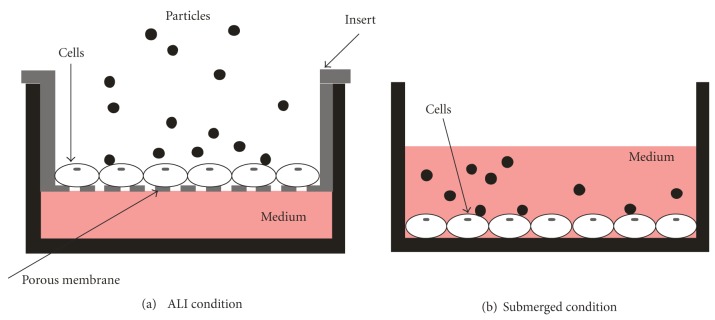
Schematic of the two cell exposure models used for studying particle-cell interaction. (a) Exposure at the air-liquid interface (ALI): airborne particles are directly deposited on cells grown at the air-liquid interface. (b) Exposure under submerged conditions: particles were suspended directly in the cell culture medium covering the cells.

Cell-based *in vitro* toxicity assays are widely used to assess the toxicity of nanoparticles. These toxicological *in vitro *studies are typically performed using cell cultures grown under submerged conditions, where the toxin/stressor is dissolved or suspended (nonsoluble nanoparticles) directly in the cell culture medium covering the cells. While this approach is experimentally simple, submerged cell exposures have two main limitations. First, the particle dose interacting with the cells is typically unknown since the particle fraction reaching the cells can neither be readily measured nor always be calculated from the hydrodynamic properties of the particles (size, density, shape) [19]. This problem is especially pronounced for particles smaller than about 100 nm, when diffusion becomes the dominant transport mechanism [20], leading to loss of particles to lateral walls. The second limitation is that submerged cell-culture conditions represent an unrealistic and artificial environment for alveolar epithelial cells in the lungs. *In vivo* exposure through inhalation involves deposition of PM onto the lung epithelium, that is, the cells are exposed to inhaled air (airborne PM) from one side while being in contact with the blood circulation from the other side. Since submerged cell systems are completely covered with cell culture medium (see Figure 1(b)), *in vivo* exposure conditions can be mimicked more realistically by exposing epithelial cells at the air-liquid interface (ALI) (Figure 1(a)). Various ALI exposure systems have been introduced [21–28], but it is unclear whether the enhanced experimental complexity of the ALI exposures compared to submerged exposures is justified. For that reason, we compared the cellular response to nanoparticles after ALI and submerged exposure. 

One of the most widely accepted paradigms of particle toxicity states that particles induce inflammation via oxidative stress and subsequent activation of redox-sensitive transcription factors [29]. Nel and colleagues refined and expanded this concept into the hierarchical oxidative stress paradigm [30, 31] suggesting the transition from an antioxidant defense response (tier1) to inflammation (tier2) and finally to cytotoxicity (tier3), if the induced stress is strong enough. Proinflammatory responses mediated by oxidative stress have been proposed to be not only crucial but also the most sensitive readout for particle toxicity [30]. We therefore measured three proinflammatory cytokines (interleukin-8 (IL-8), IL-6, and granulocyte macrophage colony-stimulating factor (GM-CSF)) and three oxidative stress markers (heme oxygenase 1 (HMOX1), superoxide dismutase (SOD-2), and glutamate-cysteine synthetase, catalytic subunit (GCS)) by qRT-PCR. 

In this study the first ALI exposure of human epithelial-like cells (A549) to airborne agglomerates of ZnO nanoparticles is presented. The dose- and time-dependent cellular responses of the cells were compared after ZnO exposure under submerged and ALI conditions at two dose levels (0.7 and 2.5 *μ*g/cm^2^) and two time points (0 h or 2 h after incubation). From the exposure-specific *in vitro* toxicity data, we deduced corresponding lowest observed effect levels (LOELs) and compared them with similar studies available in the literature. 

## 2. Materials and Methods

### 2.1. Materials

Common laboratory chemicals were purchased from Sigma-Aldrich (Taufkirchen, Germany). The particle exposure experiments were performed with commercially available powder of ZnO nanoparticles (NPs) (Alfa Aesar, Ward Hill, MA, USA ID 43141) with primary particle diameters between 24 and 71 nm (manufacturer information) and a measured BET surface area of 13 ± 2 m²/g, which agrees with the manufacturer specifications (15–45 m²/g) within experimental uncertainties.

### 2.2. Cell Culture

In this study, the alveolar epithelial-like cell line (A549) from a human lung adenocarcinoma (obtained from ATTC, Manassas, VA, USA) representing the alveolar type II phenotype [32] was used.

For ALI exposure (Figure 1(a)), A549 cells were seeded on perforated Anodisc membranes (Whatman, Maidstone, UK; aluminum oxide, diameter: 47 mm, pore size: 0.2 *μ*m) with about 1.6 × 10^5^/cm² cells and cultivated in 25 mL Petri dishes under submerged conditions for 9 d at 37°C in DMEM/F12/L-Glut/15 mM HEPES buffer (Invitrogen, Germany) containing 100 U/mL penicillin, 100 *μ*g/mL streptomycin, and 10% FCS. After 9 d a confluent layer with a cell density of approximately 5.1 × 10^5^/cm^2^ was obtained. 1 hour prior to particle exposure, the cells were transferred to the ALI, by taking the six cell-covered membranes from the Petri dishes and placing them in two cell exposure chambers (described below) using the same culture medium as above but without FCS. This arrangement allows nourishment of the cells with a cell culture medium through the perforated membrane from the bottom and exposure to airborne particles from the top. Immediately after ALI exposure, the cells were washed with PBS and gently scrapped off the membranes after adding trypsin/EDTA (for RT-PCR). For reasons discussed below, one of the cell-covered membranes in each ALI exposure chamber was incubated for a postincubation period of 2 h (submerged in 3 mL medium at 37°C) prior to determination of the biological endpoints. 

For exposure under submerged conditions (Figure 1(b)), A549 cells were seeded at 2.5 × 10^5^/cm^2^ in 24-well plates and incubated for 16 h in DMEM/F12/L-Glut/15 mM HEPES buffer (Invitrogen, Germany) containing 100 U/mL penicillin, 100 *μ*g/mL streptomycin, and 10% fetal calf serum (FCS) resulting in a cell density of approximately 3.5 × 10^5^/cm^2^. 

### 2.3. Exposure at the ALI

The ZnO powder was aerosolized with a commercially available venturi-type dry powder disperser (Model SAG 410, TOPAS, Leipzig, Germany) optimized for output stability by taking the following measures: (i) the metal venturi nozzle was replaced by a ceramic nozzle to avoid chemical and mechanical erosion, (ii) the particle reservoir and the inlet of the venturi nozzle were permanently flushed with dry nitrogen instead of filtered ambient air to minimize clogging due to moisture effects, (iii) the scraper in the reservoir was modified to allow for permanent stirring of the powder especially at the bottom of the reservoir, (iv) the aerosol output was passed through a buffer volume to remove extremely large particles (sedimentation) and smoothen fluctuation in ZnO NP concentration, and (v) particle growth due to coagulation was minimized by diluting the aerosol (1 : 1) with compressed filtered air directly after generation. 

 A detailed description of the ALI exposure chamber used here was provided by Bitterle et al. [21]. Briefly, ZnO aerosol was generated at a flow rate of 1.5 L/min with the generator described above and evenly distributed to two cell exposure chambers (one for particle exposure or control) holding three cell-covered Anodisc membranes each. The two chambers were operated in parallel using symmetric flow splitters with the control atmosphere (clean air) being obtained by filtration with a PALL filter (BB50TE, PALL, Newquay, UK). Each chamber was supplied with 0.25 L/min aerosol-laden air (or filtered air for control), which was directed at a radially symmetric stagnation point flow profile over the cell-covered membrane. This design assures spatially uniform particle deposition onto the cells at a deposition fraction, which was experimentally determined to be almost constant (2% of the particles in the sample flow) over a broad particle size range of about 50 to 500 nm [33], due to the compensating effects of diffusional and gravitational deposition [34]. The air flow was conditioned to 37°C and 99.5% relative humidity. A more detailed description of the ALI exposure chamber is provided by Bitterle et al. [21].

The particle number size distribution was measured immediately downstream of the exposure units with a scanning mobility particle sizer (SMPS, model 3080, TSI, St. Paul, MN, USA, combined with a TSI model 3025A condensation particle counter). By maintaining a constant particle concentration (within about ±20%) during the 3 h exposure time, the cell-delivered particle dose increased linearly with time. After 3 h the final dose was reached and the biological parameters were evaluated at this time point (referred to as 0 h) and after an additional 2-hour postincubation time at 37°C under submerged conditions (referred to as 2 h).

### 2.4. Exposures under Submerged Conditions

For ZnO exposures under submerged conditions, the culture medium in each well was replaced with serum-free medium into which NPs of 0.7 and 2.5 *μ*g/cm^2^ of well area were given by adding the appropriate volume of a freshly prepared 1 mg ZnO/mL H_2_O stock suspension (vortexed and sonicated twice for 1 min intermittently immediately prior to application). The size distribution of the ZnO NPs in suspension was determined with a dynamic light scattering sizer (DLSS) (HPPS 5001 Malvern Instruments Ltd., Worcestershire, UK). As shown in the Results section, gravitational settling was sufficient for all particles to reach the cells within 1 h. Thus, the final particle dose was delivered to the cells after a 1 h exposure time. The biological parameters are reported relative to control conditions (incubated cell cultures without ZnO) either directly after the exposure time (referred to as 0 h) or after an additional 2 h postincubation time (referred to as 2 h).

### 2.5. qRT-PCR Measurements of Proinflammatory and Oxidative Stress Markers (mRNA Expression)

Gene expression levels of interleukin-8 (IL-8), IL-6, granulocyte macrophage colony-stimulating factor (GM-CSF), the antioxidant enzyme heme oxygenase 1 (HMOX1), superoxide dismutase (SOD-2), and glutamate-cysteine synthetase, catalytic subunit (GCS), were measured by RT-PCR with SYBR green. After exposure, cells were lysed and homogenized in a buffer containing guanidine isothiocyanate and total RNA was isolated using a RNeasy kit according to the method recommended by the manufacturer (Quiagen, Germany). To detect cytokine mRNA expression, RNA was reverse-transcribed into cDNA using the First-Strand cDNA Kit (Pharmacia, Germany). For PCR amplification, the above-mentioned cDNA served as template and 3 *μ*L was added together with the specific 5′ and 3′ primers to the Absolute QPCR SYBR Green Mixes from ABgene (Thermo Fisher Scientific, Germany). Quantitative PCR was performed in a *Taq*Man instrument (*Taq*Man ABI Prism 7700 Sequence Detector System; Perkin-Elmer, Germany) offering the advantage of fast and real-time measurement of fluorescent signals during amplification. The housekeeping gene glyceraldehyde-3-phosphate dehydrogenase (GAPDH) was used as internal reference to normalize the RNA levels of the genes being studied. The following primers were used (sense; antisense): IL-8 (ATGACTTCCAAGCTGGCCGTGGCT; TCTCAGCCCTCTTCAAAAACTTCTC), IL-6 (GACAGCCACTCACCTCTTC; CCAGGCAAGTCTCCTCAT), GM-CSF (CTTCCTGTGCAACCCAGATT; CTTGGTCCCTCCAAGATGAC), HMOX1 (AAGATTGCCCAGAAAGCCCTGGAC; AACTGTCGCCACCAGAAAGCTGAG), SOD-2 (CCTGGAACCTCACATCAACG; AACCTGAGCCTTGGACACC), GCS (GTTCTTGAAACTCTGCAAGAGAAG; ATGGAGATGGTGTATTCTTGTCC), GAPDH (CCATGAGAAGTATGACAACAGCC; TGGCAGGTTTTTCTAGACGG).

### 2.6. Viability Assay

Cell viability was measured with the cell proliferation reagent WST-1 (Roche Applied Sciences, Germany). The WST-1 reagent is a ready-to-use solution which was added to the cells at a concentration of 100 *μ*L/mL. Light absorbance was measured after 30 min incubation at 37°C at 450 nm (iEMS Reader MF, Lab Systems).

### 2.7. Statistical Analysis

Results are presented as geometric mean and geometric standard error of the mean of at least four separate experiments (*n* = 4–7), since the data are not normally but close to log normally distributed. Data comparisons were carried out using the Kruskal Wallis test (Statgraphics plus 5.0), a nonparametric one-way analysis of variance (ANOVA). *P* < 0.05 was considered as statistically significant.

## 3. Results

### 3.1. Particle Size Distribution

**Figure 2:**
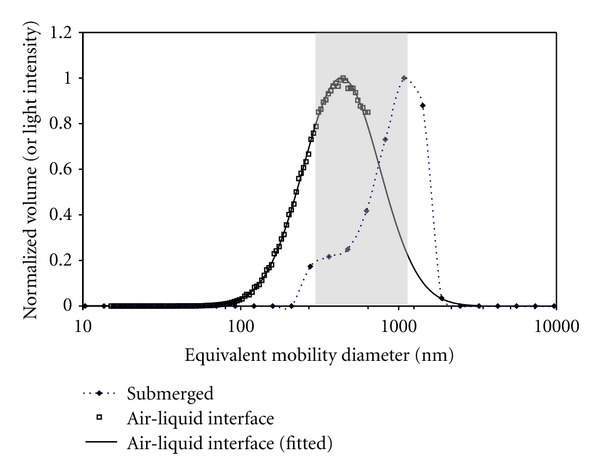
Typical ZnO particle size distribution during ALI submerged (SUB) exposure conditions, respectively. When comparing the particle size distributions during ALI and submerged exposures, one has to consider that different sizing instruments were used. As discussed in the text, both SMPS (ALI) and DLSS (submerged) measure the particle mobility diameter. Furthermore, the volume distribution (normalized to the maximum volume level) is approximately equal to the (normalized) light intensity distribution in the size range between about 315 to 1250 nm (highlighted by the grey shaded area), which encompasses most of the size regime of interest here. The ALI size distributions showed volume-weighted median diameters of about 335 ± 40 nm and a width of 1.77 ± 0.05 (geometric standard deviations). For the submerged conditions (SUB), dynamic light scattering measurements (DLSS) showed ZnO aggregates of about 900 nm (mobility diameter) with a less pronounced (~20%) secondary mode near 350 nm. Thus it is evident that the average ZnO agglomerates were considerably larger during submerged than during ALI exposure condition. For comparison with other studies, the number-weighted size distribution of the ZnO particles during ALI exposure had a count median diameter of about 140 nm (data not shown).

The measurement of particle size distributions in different media, such as air and liquid during ALI and submerged exposure, respectively, requires the use of different measurement techniques, here scanning mobility sizing with an SMPS (ALI) and dynamic light scattering using a DLSS (submerged). The SMPS counts individual particles, which are size-selected based on their migration speed in an electric field [35]. The DLSS determines the mobility diameter from the time-dependent fluctuations of the scattered light intensity signal from an ensemble of suspended particles [36]. While both instruments measure the mobility diameter (*x*-axis of size distribution as depicted in Figure 2), the SMPS counts individual particles and the DLSS reports a signal proportional to the light intensity of a given particle [35]. Since the light intensity signal cannot be directly related to particle number concentration, the SMPS number distribution was converted into effective volume (or mass) distribution, which can be related to the scattered light intensity as described below. Accurate performance and comparability of both instruments was validated with NIST-traceable (National Institute of Standards and Technology, USA) reference particles.

 For ALI exposures, the SMPS measurements of the ZnO aerosol revealed a count median (mobility) diameter (CMD) and geometric standard deviation of 141 ± 12 nm and 1.77 ± 0.05, respectively. Since the CMD is larger than the diameter of the primary ZnO NPs (24–71 nm), it is evident that the ZnO aerosol mainly consists of agglomerated (nonspherical) structures. For the two dose levels studied here, the mean and standard deviation of the number concentration was (3.5 ± 0.45) × 10^5^ and (9.5 ± 0.9) × 10^5^ particles/cm^3^. This corresponds to average mass concentrations of 10.1 mg/m^3^ and 30.4 mg/m^3^, respectively, with an almost constant mass (or volume) median diameter (±standard deviation) of 335 ± 40 nm. As mentioned above, for comparison of SMPS and DLSS, data the SMPS data were converted from number- into mass-based (volume-based) size distribution taking into account the nonspherical shape of the ZnO particles as follows. First, the number size distribution was converted into volume distribution (assuming spherical particle shape for now) and fitted as lognormal distribution using the Hatch-Choate equations for consistent conversion of the count median into mass median diameter. Integration of the volume distribution yields the total volume. For correct conversion into particle mass accounting for the nonspherical particle shape, the volume is multiplied by the effective density of 4.6 g/cm^3^ [37], which was experimentally determined by dividing the gravimetrically determined particle mass by the (spherical) particle volume determined from the SMPS data (see Figure 2). The fact that the effective density of the ZnO aerosol is smaller than the bulk density of ZnO (5.6 g/cm^3^) is consistent with the agglomerated structure of the ZnO particles [37]. The relatively small difference between effective and bulk density indicates that the particles have a relatively compact (sphere-like) structure.

Although exposures under submerged conditions were performed with the same ZnO particles as used for ALI exposures, ZnO particles suspended in the cell culture medium were more agglomerated and hence larger than those dispersed in air (ALI exposure). As seen from the DLSS size distribution depicted in Figure 2, the ZnO particles, suspended in cell medium for 30 min, displayed a minor mode near 350 nm (about 20% of total mass) and a more pronounced mode near 900 nm (~80% of total particle mass). For accurate comparison of the DLSS sizing data, one has to relate the volume-based size distribution derived from the SMPS data with the light intensity values of the DLSS. For particle sizes near the wavelength *λ* of the light source (between about *λ*/2 and 2*λ*), it has been shown that the light-intensity-to-volume ratio is almost constant [38]. Thus for the Malvern DLSS (*λ* = 633 nm), we can assume that the normalized light intensity distribution (normalized to the maximum of the intensity spectrum) as shown in Figure 2 is approximately equal to the normalized volume (or mass) distribution obtained from the SMPS in the size range between 315 and 1250 nm (shaded area in Figure 2), which covers most of the size range of interest for the present study. Outside this range, the light intensity level is systematically lower than the corresponding volume level [38]. 

In summary, it is evident that the ALI size distribution was dominated by ZnO agglomerates with a volume median diameter near 350 nm. While this mode is also seen during submerged exposures, most of the particle mass (~80%) resides in a mode near 900 nm indicating that suspending the ZnO particles in cell culture medium for 30 min leads to enhanced particle size due to agglomeration effects.

### 3.2. Particle Dosimetry

For reliable comparison of the cellular dose-response relationship under ALI and submerged culture conditions, the biological response should be correlated to the cell-delivered particle dose (*D*_*M*_) normalized to the cell-covered surface area. Here *D*_*M*_ is given by

(1) $$\begin{array}{c} D_{M}=\frac{M\;\text{Dep}}{A_{\text{cell}}}, \end{array}$$

where *M* is the particle mass passing or floating over the cell layer during the exposure, Dep is the deposition efficiency (fraction of particles depositing onto the cell layer), and *A*_cell_ is the area covered by the exposed cells.

 For the ALI exposure, *M* is calculated from *M* = *mQt*, where *m* is the average mass concentration (ZnO mass per volume air; 10.1 and 30.4 mg/m^3^ for the low and high dose level, resp.), *Q* = 0.25 L/min is the volumetric flow rate passing over the cell layer, and *t* = 3 h is the exposure time. With *A*_cell_ = 12.6 cm^2^ (per culture membrane) and Dep = 0.02 [21, 33], we find from (1) that *D*_*M*,ALI_ = 0.7 and 2.2 *μ*g ZnO/cm^2^ for the low and high doses, respectively. With the specific BET surface area of 13 m^2^/g, this corresponds to BET surface area doses of 0.09 and 0.29 cm^2^ ZnO/cm^2^, respectively. 

For submerged exposures, we substitute *M* by *M* = *mV* in (1), where *m* is the ZnO particle mass concentration in the stock suspension (here 1 mg/mL) and *V* is the volume of the ZnO stock suspension (here 1.4 or 5 *μ*L) added to the culture medium (1 mL). Under the assumption that all particles contained in the medium will deposit on the cells within 3 h (i.e., Dep = 1; will be justified below) we find from (1) that *D*_*M*,sub_ = 0.7 and 2.5 *μ*g ZnO/cm^2^ (with *A*_cell_ = 2.0 cm^2^) or 0.09 and 0.33 cm^2^ ZnO/cm^2^, respectively.

As a justification for Dep = 1 under submerged conditions, the following aspects were considered [39]: (I) Is the particle deposition dominated by sedimentation or diffusion (the latter would result in loss of particles to the lateral walls and hence Dep < 1) and (II) if sedimentation dominates particle deposition, is the exposure time long enough for all particles (even the ones near the top of the cell culture well) to reach the cells at the bottom of the well? To address these issues, we calculated the gravitational settling velocity and the mean diffusional displacement speed of the particles in water to be 56 mm/h and 0.033 mm/h, respectively [20], where we assumed an average particle diameter of 900 nm (see Figure 2). Since the ratio of gravitational to diffusional displacement speed is about 1700 for 900 nm particles with a density of 4.6 g/cm^3^ (ZnO), sedimentation is the dominant deposition mechanism; that is, negligible particle loss to lateral walls is expected. Secondly, for a sedimentation speed of 56 mm/h and a 50 mm depth of the cell culture medium (1 mL of medium; 2 cm^2^ cross-sectional area of well), all ZnO particles are expected to deposit onto the cells within about 1 h. 

As a caveat we note that due to differences in deposition kinetics during ALI and submerged exposures (as described above), the final dose was delivered to the cells after 3 h and 1 h, respectively.

### 3.3. Biological Endpoints

Several biological endpoints were investigated after ZnO exposures of the A549 cells representing human alveolar epithelium type II cells [32, 40]. First, cell viability was determined to exclude the possibility that the observed effects of ZnO NPs on gene expression levels are negatively biased due to cytotoxic effects. No effects on cell viability were seen for the ZnO concentrations investigated here for both ALI (viability in % of (submerged) unchallenged control: 93.5% ± 2.1, at ≤2.2 *μ*g/cm²) and submerged conditions (94% ± 7.99, at ≤2.5 *μ*g/cm²). Hence, the cellular response to ZnO exposure was not significantly hampered by reduced cell viability and there was no significant reduction in cell viability due to exposure of the cells to the air-liquid interface. 

**Figure 3:**
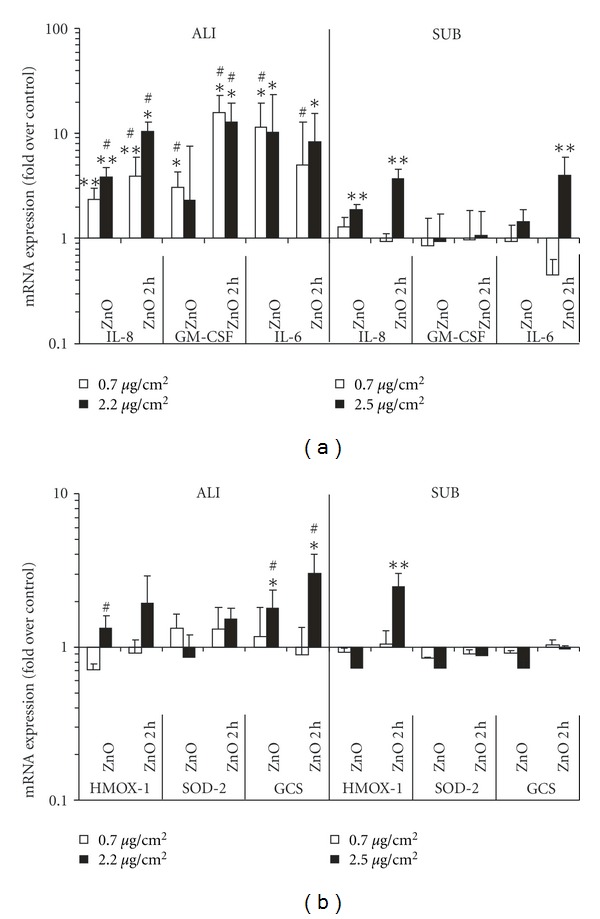
Comparison of the effect of ZnO on proinflammatory and oxidative stress markers in A549 cells following exposure at the ALI and under submerged (SUB) conditions. (a) mRNA expression of proinflammatory cytokines (IL-8, GM-CSF, and IL-6) was measured with RT-PCR either directly after (0 h after incubation) or two hours after the exposure (2 h). (b) Same as (a), but for oxidative stress markers (HMOX1, SOD-2, and GCS). The postincubation of the cells after ALI exposure was also performed under submerged conditions. The mRNA values were normalized to glyceraldehyde-3-phosphate dehydrogenase (GAPDH) levels and expressed as the fold increase over control (the control level was set to unity) which was filtered air and pure medium for ALI and SUB, respectively. The data show the geometric means and geometric standard error of the mean based on 4 to 7 independent experiments. Due to differences in the deposition kinetics described in the experimental section, the final dose was delivered to the cells after 3 h (ALI, open bars 0.7 *μ*g/cm^2^ and solid bars 2.2 *μ*g/cm^2^) or 1 h (submerged, open bars 0.7 *μ*g/cm^2^ and solid bars 2.5 *μ*g/cm²). The symbol (∗) indicates significant differences from control levels at *P* < 0.05, and (∗∗) at *P* < 0.01. The symbol (#) indicates mRNA values which are statistically different from the corresponding submerged mRNA levels (differences are 2.8 to 12-fold (*P* > 0.05)).

ALI exposure of A549 cells to ZnO NPs caused elevated levels of mRNA coding for IL-8, GM-CSF, and IL-6 as shown in Figure 3(a) (left panel). IL-8 showed a significant increase with increasing dose and time. The time response of GM-CSF was similar to that of IL-8, but no significant dose effect was observed. IL-6 was increased for all ALI exposure scenarios, but no significant dependence on dose or time was observed. On the other hand, the oxidative stress markers HMOX1 and SOD-2 showed no significant increases in mRNA expression except for GCS mRNA which was slightly, but statistically significantly increased for both time points of the high dose level (1.8-fold and 3-fold increased at 0 h and 2 h, resp., see Figure 3(b), left panel).

Under submerged conditions, the expression levels of all proinflammatory markers were lower than those under ALI conditions (Figure 3(a), right panel). For IL-8 only the high dose showed a significant induction of 1.9-fold and 3.7-fold at the two time points, and IL-6 was increased (4-fold) for the high dose at 2 h. Out of the three oxidative stress markers, a significant expression was observed only for HMOX1 (2.7-fold) after 2 h (Figure 3(b), right panel).

In summary, compared to the submerged conditions, ALI exposure showed slight, but statistically significant enhancements in mRNA expression of IL-8, GM-CSF, and IL-6 for all dose levels and time points as well as for the high dose level of GCS (both time points). The only case where the submerged exceeded the ALI response was HMOX1 (high dose, 2 h). Thus the ALI exposure system was generally more sensitive to mRNA induction than the submerged exposure assay especially for the proinflammatory markers. 

## 4. Discussion

To the best of our knowledge, the data presented here represents the first *in vitro *measurements of the cellular response to an exposure of airborne agglomerates of ZnO particles at the ALI. We model inhalation exposure to ZnO NPs with the widely used A549 cell line. A549 cells represent human alveolar epithelial type II cells [32, 40], which are considered the defenders of the alveoli because they are important producers of cytokines [41] and metabolically more active than type I pneumocytes [42, 43]. Consequently, A549 cells are widely regarded as a valid model cell system for pulmonary particle toxicity studies [44, 45]. 

The data presented here are consistent with positive dose-response correlations. Focusing on ALI conditions first, we find that the IL-8 response (2 h postincubation time) is enhanced for the higher concentration with a 94.5% confidence level (*P* = 0.055). A positive dose-response is also found for GCS (2 h postincubation time; 95% confidence level). On the other hand, no significant response is found for HMOX1 and SOD2 for any of the concentrations used here, since none of these parameters is upregulated. Furthermore, the lack of a dose-response correlation for GM-CSF and IL-6 might be due to reaching the saturation levels already for the lower concentration. Similar considerations can be conducted for the submerged cell culture data. A positive dose-response is seen for IL-8 (both time points), IL-6, and HMOX-1 after 2 h postincubation time. All other parameters are not changed.

While historically *in vitro* particle toxicity studies have been performed with submerged cell systems, various ALI exposure systems have recently been introduced in an attempt to mimic more realistically the exposure conditions during particle inhalation [21–28]. Further advantages of ALI exposures include the preservation of the physicochemical characteristics of the airborne particles (e.g., particle agglomeration and/or particle-medium interactions such as partial dissolution of ZnO in cell culture medium are avoided [46]), the synergistic effects between particulate and gaseous compounds can be investigated (e.g., relevant for combustion emissions) and the biological complexity can be more adequately represented (e.g., surfactant coating can be added to of alveolar epithelial cells). Last but not least, it is typically technically simpler to determine the cell-delivered particle dose under ALI than submerged conditions [47, 48]. Some of the recently introduced ALI exposure systems utilize aerosolized nanoparticle suspensions instead of dry airborne nanoparticles [28]. While these systems allow for cell exposure at the ALI, partial dissolution and possibly agglomeration of the nanoparticles cannot be ruled out with these systems. 

Although ALI exposures have become more widely used, there is very little quantitative information on whether and how the exposure type (ALI and submerged) affects the cellular response. A summary of the currently available studies is listed in Table 1 and will be discussed below. In Table 1 all but one investigator utilizes gene expression analysis instead of protein determination as toxicological readout. Gene expression is commonly preceding protein expression; however, the latter can additionally be regulated at the posttranscriptional level. In some cases, protein expression without associated gene expression can occur. However, this is not the case for any of the markers listed in Table 1. Hence, both protein and gene expressions are suitable for toxicity studies. The advantages of gene expression analysis by qPCR include higher sensitivity than the measurement of protein levels, simultaneous quantification of several markers and higher cost efficiency. For these reasons, gene expression analysis was used in the present study to screen for representative markers of different acute response pathways related to inflammation and oxidative stress.

As mentioned above, the cellular response to nanoparticles depends on numerous aspects including cell type, pre- and postprocessing of the cells, state of cell differentiation, particle dose, deposition kinetics, and physicochemical particle characteristics. Matching all of these aspects is very difficult, if not impossible, since, for instance, the state of cell differentiation is inherently different under submerged and ALI culture conditions [49–51] and particle deposition rates onto the cell system may vary significantly for submerged and ALI conditions, since they depend on agglomeration state and carrier medium of the particles (air or liquid). Hence, any study on cellular response under ALI versus submerged cell conditions should provide as much details on these aspects as possible as is done in the following section. 

Exposures were performed at two dose levels with no statistically significant difference for the two exposure types (low: 0.7 *μ*g/cm²; high: 2.2 *μ*g/cm² (ALI) and 2.5 *μ*g/cm² (submerged)). In addition, the cell type (A549) was identical for both exposure types and the cell densities (cells per cm²) were similar at the time of exposure. Preprocessing of the cells was different because it had to be adapted to the two different exposure conditions, whereas postprocessing of the cells was identical. There have been several studies indicating differences in cell differentiation due to transfer of the cells from submerged to ALI culture conditions even at the first investigated time points between a few hours and 1 d [49–51]. However, we assume that in the present study the state of cell differentiation was similar, since cells were kept under submerged conditions except for a brief period of time during ALI exposure (1 h prior to exposure; 3 h during exposure). This is supported by the fact that we found no statistically significant differences in cell viability as well as IL-8 mRNA and HMOX-1 mRNA after transfer of the cells to ALI conditions. This is an important aspect, since with an already strained antioxidant defense, as, for instance, reported by [49], cells may be more susceptible to the effects of the particle exposure. Other important aspects for ALI-submerged comparisons are related to the particle characteristics. For ALI conditions, the count median and mass median particle diameters were 141 nm and 335 nm, respectively, and the rate of particle deposition onto the cells was constant (within 20%) during the 3 h exposure time by keeping the sample flow and the ZnO aerosol concentration constant. Under submerged conditions, the size of the ZnO particles increased from a mass median diameter of about 350 nm to about 900 nm within about 30 min due to agglomeration, which results in an approximately 3-fold deposition rate; that is, the entire ZnO dose is delivered to the cells with about 1 h. Hence, differences in deposition kinetics may affect the comparison of the two exposure scenarios. Since the total number of primary particles in the medium is not changing with the agglomeration state, agglomeration does not affect the number of primary particles or the surface area dose delivered to the cells. However, agglomerate size may influence the biological response of the A549. Furthermore, ZnO is partially soluble in aqueous media [30]. Hence, the Zn^2+^/ZnO ratio may be different under ALI and submerged conditions with higher Zn^2+^/ZnO ratios to be expected under submerged conditions due to the relatively high dissolution of ZnO in the cell culture medium. 

 In spite of some experimental differences between submerged and ALI exposures, as described above, it is instructive to compare the ZnO dose-response curves observed under ALI and submerged conditions and relate these findings to similar data sets for other particle and pulmonary cell types from the literature. This can be done by determining the dose range in which the lowest observed effect levels (LOELs) occurred. If none of the two dose levels investigated here showed a statistically significant response, the LOEL lies above the highest dose level (>2.5 *μ*g/cm^2^). If the low dose showed no response, but the high dose did, then the LOEL falls in the range of 0.7–2.5 *μ*g/cm^2^. If both dose levels showed a response, then the LOEL is below <0.7 *μ*g/cm^2^. 

As seen from Table 1, our data indicate that four biological parameters (mRNA levels of IL-8, GM-CSF, IL-6, and GCS) showed lower LOELs and hence elevated response levels under ALI conditions. The results for two of the six investigated parameters (HMOX1, SOD-2) were inconclusive, since the investigated dose regime was not broad enough to discern differences in LOEL. Similar results were reported by other studies with pulmonary cell lines and primary cells reported in the literature (Table 1). Volckens and colleagues [26] exposed primary human bronchial epithelial cells to concentrated coarse ambient particulate matter. Applying our LOEL scheme to their data indicates that the mRNA levels of IL-8 and HMOX1 were more pronounced under ALI conditions, while no conclusive result was found for COX-2 mRNA expression. Holder and colleagues [24] investigated Diesel exhaust particles with a human bronchial epithelial cell line (16HGE14o). As seen from Table 1, they found no conclusive result for IL-8 protein levels but state that a much smaller dose was required to induce similar IL-8 expression levels. We contend that this claim is not substantiated by their data, since in contrast to submerged exposures their IL-8 response under ALI conditions was not statistically significantly different from unity (according to their own statistical analysis). It is important to note that the currently available data on submerged versus air-liquid exposure comparisons is limited, but diverse. As seen from Table 1 the data were generated with both immortalized and primary cell cultures as well as with different particle types, various biological endpoints, and different pre-/postprocessing protocols. These differences are likely to result in exposure-dependent differences in the state of cell differentiation, particle-cell interaction, and deposition kinetics. In spite of this heterogeneity, none of the currently available studies has identified a biological parameter, which responded to be more sensitive to particle challenge under submerged exposure conditions than under ALI conditions. While it cannot be inferred that this is true for all possible biological endpoints, the currently available data suggest that air-liquid interface exposures are a more “conservative” toxicity test than submerged systems, that is, ALI cell systems are likely to lead to less false negatives.

To put the particle dose levels typically used for *in vitro* toxicity testing into perspective, it is instructive to consider that the currently recommended Occupational Safety and Health Administration (OSHA) standard for ZnO fume (and many other occupational dusts) is 5 mg of ZnO fume per cubic meter of air (mg/m^3^) averaged over an eight-hour-per-day work shift. Assuming an accumulated breathing volume of 3 m^3^ in 8 h, a lung surface area of 140 m^2^, an alveolar deposition efficiency of 10–50% depending on particle size, and negligible clearance from the alveolar regime within 24 h [52], the OSHA standard corresponds to a daily alveolar surface dose of 1.1–5.4 ng/cm², which is about 3 orders of magnitudes smaller than what was deposited during the ZnO ALI exposures performed here (0.7–2.2 *μ*g/cm^2^). Furthermore, it can be seen from Table 1 that the LOEL during submerged exposures is typically between 1 and 65 *μ*g/cm^2^, which is in the range of the expected lifetime dose (4–18 *μ*g/cm^2^) under worst case conditions represented by a worker exposed to the OSHA (ZnO) dust limit (5 mg/m^3^) for 5 days per week, 50 weeks per year for 45 years (where we assumed that only about 30% of the lung-deposited particles remain in the alveoli due to alveolar clearance mechanisms). As the *in vitro* dose is deposited onto the cells within a few hours instead of 45 years, this does not represent a realistic *in vivo* exposure scenario. In spite of these unrealistically high dose levels, *in vitro* cell tests are useful for pharmacological and toxicological prescreening of substances and studies of cellular response mechanisms, but lower cellular doses may be desirable. Table 1 suggests that an incremental progress may be possible with ALI cell systems. In combination with other measures such as the use of multicell cocultures instead of single-cell cultures, this may lead to significantly more realistic *in vitro* dose rates in the future.

## 5. Conclusion

In this study the *in vitro* response of pulmonary epithelial cells to different types of (nano-)particles was compared for air-liquid interface (ALI) and submerged exposure conditions. The scarce data pool on this issue was expanded by presenting the first ALI data on airborne agglomerates of ZnO nanoparticles using alveolar epithelial-like type II cells (A549). For ZnO, the lowest observed effect levels (LOELs) of the proinflammatory markers (mRNA gene expression of IL-8, IL-6, and GM-CSF) were lower under ALI than under submerged conditions, while no significant response was observed for most of the oxidative stress markers (HMOX1, SOD-2, and GCS). These findings are consistent with the few previous comparative studies on this issue indicating that toxicity testing with the conventional submerged systems may yield more false negatives than the more recently developed ALI systems. 

The dose levels used here and in similar studies reported in the literature are in the range of an entire lifetime dose of occupational dust received by a heavily exposed worker. The ability to induce cellular responses at somewhat lower and hence more realistic dose levels under ALI conditions may provide biologically more meaningful data than those obtainable with the conventional submerged exposures. Further advantages of ALI cell systems include the biologically more realistic exposure scenario (cells in the lungs are exposed under ALI-like not submerged conditions), the absence of inadvertent modifications of the particle properties in the cell culture medium (e.g., agglomeration, partial dissolution), and the possibility of direct dose measurement (e.g., quartz crystal microbalance). Depending on the application, these aspects may outweigh the larger experimental complexity of ALI exposures. However, quantitative comparisons of the cellular response under ALI and submerged culture conditions are still very limited. Thus, further studies are needed to address these issues.

## Figures and Tables

**Table 1:** Comparison of the lowest observed effect levels (LOELs) for nanoparticles exposure of cells exposed at ALI and submerged conditions.

Reference	Particle type	Human epithelial cell type	Time at ALI prior to exposure	Exposure time	Postincubation time	Exposure time	Postincubation time	Biological parameter	Dose for the lowest observed effect level (LOEL)^1^		
				ALI	ALI	Submerged	Submerged		Submerged (*μ*g/cm^2^)	ALI (*μ*g/cm^2^)	ALI more sensitive than submerged
								IL-8 (mRNA)	12.5–25	≤2.0	yes
[26]	Coarse urban PM	Bronchial (primary)	3 d	3 h	0 h	0 h	1 h	HOX-1 (mRNA)	25–65	≤2.0	yes
								COX-2 (mRNA)	≤7.0	≤2.0	unclear

[24]	Diesel soot	Bronchial (16HBE14o)	?^2^	6 h	20 h	6 h	20 h	IL-8 (protein)	0.25–1.88	>0.1	unclear

								IL-8 (mRNA)	0.7–2.5	<0.7	yes
								GM-CSF (mRNA)	>2.5	0.7^3^	yes
This study	Agglomerated ZnO nanoparticles	Alveolar type II (A549)	1 h	3 h	0 h	1 h	0 h	IL-6 (mRNA)	>2.5	<0.7	yes
								HMOX1 (mRNA)	>2.5	>2.2	unclear
								SOD-2 (mRNA)	>2.5	>2.2	unclear
								GCS (mRNA)	>2.5	0.7–2.5	yes

^1^LOEL: lowest dose in *μ*g/cm^2^ at which a statistically significant effect (*P* < 0.05) was observed relative to the control.

^2^Not stated in publication.

^3^Here: 0.7 *μ*g/cm^2^ showed a significant effect, while 2.2 *μ*g/cm^2^ was not statistically significant due to larger variability in the data. Hence, we assume that 0.7 *μ*g/cm^2^ is a good estimate for LOEL.
